# Choix de carrière des étudiants en médecine ivoiriens en fin de cursus : facteurs d’influence et aspirations

**DOI:** 10.48327/mtsi.v2i1.2022.202

**Published:** 2022-02-04

**Authors:** Marie-Paule Bernadette N’CHO-MOTTOH, Iklo COULIBALY, Bénédicte BOKA, Djenamba BAMBA-KAMAGATE, Arnaud EKOU, Alain AUBREGE

**Affiliations:** 1 Institut de Cardiologie d’Abidjan, BP V 206, Abidjan, Côte-d’Ivoire; 2 Université de Lorraine, Vandœuvre-lès-Nancy, France

**Keywords:** Choix de carrière, Spécialité, Étudiants en médecine, Abidjan, Côte d’Ivoire, Afrique subsaharienne, Carrier choice, Speciality, Medical students, Abidjan, Ivory Coast, Sub-Saharan Africa

## Abstract

**Objectif:**

En Côte d’Ivoire, l’implantation des structures sanitaires pour améliorer l’accessibilité géographique aux soins reste inégale entre milieu rural et urbain. C’est dans ce contexte que l’étudiant en médecine doit se prononcer sur son choix de carrière en tenant également compte de ses préférences personnelles. Le but de notre étude était d’évaluer les aspirations et les facteurs influençant le choix de spécialité des étudiants de la faculté de médecine de l’université Félix Houphouët-Boigny d’Abidjan.

**Méthodologie:**

Les étudiants en médecine inscrits en 6e année ont rempli un questionnaire anonyme auto-administré. Il s’agissait d’un questionnaire au format papier divisé en 3 parties: critères socio-démographiques; choix de spécialité et facteurs influençant le choix de carrière. Les étudiants étaient invités à évaluer dans quelle mesure ils percevaient chacun des 24 items comme influençant leur choix de carrière au moyen d’une échelle de Likert allant de 1 (aucune influence) à 5 (grande influence). Les facteurs ont été comparés selon le choix de spécialité (médicale ou chirurgicale).

**Résultats:**

Les 3 disciplines de spécialité les plus choisies étaient: la cardiologie (17,9 %), la gynéco-obstétrique (15,7 %) et la pédiatrie (9,6 %). Le désir de passer le concours d’internat était plus fréquent chez les étudiants ayant choisi une spécialité chirurgicale (p = 0,02). Le choix d’une spécialité médicale était plus influencé par le désir d’un travail à temps partiel (p = 0,04). Les étudiants ayant choisi une spécialité médicale étaient plus guidés par l’engagement social que ceux ayant choisi une spécialité chirurgicale (p = 0,04). Par contre, ces derniers étaient plus influencés par le prestige auprès des collègues (p = 0,04) et les résultats immédiats après intervention (p = 0,01).

**Conclusion:**

L’équipement efficient des structures sanitaires pourrait contribuer à une mise en valeur des autres spécialités moins choisies en les rendant plus attractives. Une réorganisation du système avec affectation d’enseignants dans les hôpitaux régionaux avec un minimum d’équipement est indispensable afin de permettre une « décentralisation » du cursus de spécialisation, surtout pour les spécialités chirurgicales.

Quant à l’aspiration au travail à temps partiel, elle peut s’expliquer par le besoin de concilier vie familiale et vie professionnelle, mais aussi par un projet parfois inavoué de développer une activité extra-médicale lucrative en vue de compenser une faible rémunération.

## Abstract (en)

**Introduction:**

The implantation of health structures in Côte d’Ivoire to improve geographical accessibility to care remains unequal between rural and urban areas. The medical student has to decide on his or her career choice in this context, while also taking into account personal preferences. The aim of our study was to evaluate the factors influencing the speciality choice of medical students at the faculty of medicine of Félix Houphouët-Boigny University in Abidjan.

**Methodology:**

Medical students enrolled in the 6th year completed an anonymous self-administered questionnaire. The questionnaire was in paper format and was divided into three parts: socio-demographic criteria; speciality choice; and factors influencing career choice. Students were asked to rate the extent to which they perceived each of the 24 items as influencing their career choice using a Likert scale ranging from 1 (no influence) to 5 (strong influence). The factors were compared according to the speciality choice (medical or surgical).

**Results:**

The 3 most chosen specialties were: cardiology (17.9%), gynaecology-obstetrics (15.7%) and paediatrics (9.6%). The desire to take the internship competition was more frequent among students who chose a surgical speciality (p = 0.02). The choice of a medical speciality was more influenced by the willingness to work part-time (p = 0.04). Students who choose a medical speciality were more guided by social commitment than those who chose a surgical speciality (p = 0.04). In contrast, the latter were more influenced by prestige among colleagues (p = 0.04) and immediate postoperative outcomes (p = 0.01).

**Conclusion:**

The efficient equipment of health structures could contribute to the development of other less chosen specialities by making them more attractive. A reorganisation of the system with the deployment of teachers in regional hospitals with a minimum of equipment is indispensable in order to allow a “decentralization” of the specialization curriculum, especially for the surgical specialties.

As for the aspiration to part-time work, it can be explained by the need to reconcile family and professional life, but also by a sometimes unspoken project to develop a lucrative extra-medical activity in order to make up insufficient wages.

## Introduction

Selon l’Organisation mondiale de la santé, l’Afrique abrite un quart des malades dans le monde. Mais elle ne bénéficie que de 1,3 % des ressources financières mondiales consacrées à la santé, et dispose seulement de 3 % des professionnels de santé [8].

La formation et la fidélisation du personnel médical, incluant les médecins, constituent la principale difficulté. Selon la Commission « Health professionals for a new century » (Professionnels de la santé pour un siècle nouveau) du *Lancet,* ce problème est en réalité mondial. Les facultés de médecine francophones subsahariennes constituent un réseau majeur dans le cadre du dispositif de formation des professionnels de santé dans cette région d’Afrique [10].

En fin de cursus, les étudiants en médecine doivent faire des choix de spécialité qui auront un impact sur leur vie personnelle et professionnelle, mais aussi sur le système sanitaire. Les besoins en spécialistes sont variables d’un continent à l’autre. Quasiment partout en Afrique, le système de santé est organisé de manière pyramidale du point de vue spatial et fonctionnel: des structures de premier niveau (dispensaires de premiers soins, centres de santé pour les pathologies courantes, les soins de proximité et la santé maternelle); ensuite des structures de type hôpital de district ou régional de 100 à 200 lits qui normalement offrent une palette de soins de consultations externes avec de l’hospitalisation (médecine, pédiatrie, chirurgie, maternité et, parfois, service d’urgence); des structures de référence au plan national offrant les spécialités et, enfin, des centres hospitalo-universitaires qui concentrent la quasi-totalité des spécialistes, chargés d’enseignement dans les facultés de médecine [8]. Les structures de soins primaires représentent 87 % de l’offre de soins nationale à Abidjan, soit un centre de santé pour plus de 5 000 habitants et 1 médecin pour 10 000 habitants [7].

En Côte d’Ivoire, les étudiants en médecine en fin de cursus ont deux issues professionnelles: passer le concours d’internat et poursuivre une carrière hospitalo-universitaire, ou soutenir directement leur thèse d’exercice avec la possibilité de faire une spécialité. Contrairement au système français, l’internat (actuellement transformé en « épreuves classantes nationales ») n’est pas obligatoire. La seconde différence avec le système français réside dans la non-reconnaissance de la médecine générale comme spécialité. En effet, en Côte d’Ivoire, le médecin généraliste est celui qui n’a pas fait de spécialité.

L’implantation des structures sanitaires de base pour améliorer l’accessibilité géographique aux soins sur le territoire ivoirien reste inégale entre milieu rural et urbain [19]. C’est dans ce contexte que l’étudiant en médecine doit se prononcer sur son choix de carrière en tenant également compte de ses préférences personnelles.

Plusieurs auteurs, essentiellement d’Amérique du Nord ont analysé les facteurs d’influence du choix de carrière. La plupart des études ont classé les facteurs d’influence selon trois catégories: les caractéristiques socio-démographiques des étudiants, les caractéristiques des facultés, enfin l’interaction entre les caractéristiques des spécialités et les attitudes et valeurs personnelles des étudiants [15, 17]. L’approche qualitative était la plus utilisée et reposait sur des entretiens de groupe d’étudiants ou un recensement de critères sélectionnés par les étudiants au sein d’une liste [14]. En ce qui concerne les études quantitatives, Murdoch aux États-Unis en 2001 et Wright au Canada en 2004 ont validé un questionnaire en anglais d’attitudes et de valeurs des étudiants en médecine pour leur choix de carrière médicale future [13, 18]. Une version française du questionnaire de Wright élaborée par Beaulieu et al a été validée en 2010 [3]. À notre connaissance, aucune étude ne s’est intéressée aux différents critères de choix de carrière des étudiants en Afrique subsaharienne.

Le but de notre étude était d’évaluer les facteurs et les aspirations influençant le choix de spécialité des étudiants en médecine de la faculté de l’université Félix Houphouët-Boigny d’Abidjan.

## Méthodologie

Cette étude s’est déroulée à l’unité de formation et de recherche de médecine de l’Université Félix Houphouët-Boigny d’Abidjan (Côte d’Ivoire). Les étudiants en médecine inscrits en 6e année ont rempli un questionnaire anonyme auto-administré. Celui-ci a été expliqué par un enseignant. Le remplissage du questionnaire a eu lieu en avril 2021 après une épreuve d’unité d’enseignement. L’opération a été répétée lors de l’épreuve d’unité d’enseignement suivante pour les étudiants absents le premier jour du remplissage du questionnaire.

Il s’agissait d’un questionnaire au format papier divisé en 3 parties: critères socio-démographiques; choix de spécialité et facteurs influençant le choix de carrière (Annexe 1). Les étudiants étaient invités à évaluer dans quelle mesure ils percevaient chacun des 24 items comme influençant leur choix de carrière au moyen d’une échelle de Likert allant de 1 (aucune influence) à 5 (grande influence). Ces items étaient regroupés en 4 classes définies par l’étude de Beaulieu [3] et reprises intégralement: style de vie médicale, orientation sociétale, prestige, structure de soins, avec l’ajout d’une classe étudiant l’influence de la formation.

L’analyse des données a été effectuée en utilisant le logiciel SPSS version 22.0. Les variables quantitatives ayant une distribution normale étaient exprimées en moyenne écart-type et les variables catégorielles en pourcentage. Les facteurs ont été regroupés en 5 classes et ceux-ci ont été comparés selon le choix de spécialité (médicale ou chirurgicale). Les données qualitatives ont été comparées par test de Khi-deux. Les données quantitatives n’ayant pas une distribution normale ont été interprétées avec des tests non paramétriques. Un p < 0,05 était considéré comme significatif.

## Résultats

Au total 297 étudiants ont été interrogés sur un effectif de 407 inscrits en 6e année, soit un taux de participation de 72,9 %. Seuls 10 étudiants ne voulaient pas faire de spécialité après leur thèse.

L’âge moyen était de 25,5 ± 2,2 ans et il y avait 42,2 % de femmes.

Parmi les étudiants désireux de faire une spécialité (287 étudiants), 75,6 % avaient le projet de passer l’internat en vue d’une carrière universitaire. Le choix du travail à temps partiel avait été retenu chez 76,7 % des étudiants. Le niveau d’instruction des parents était très variable. Le statut non scolarisé et niveau primaire étaient plus fréquents chez les mères que chez les pères des étudiants participant à l’enquête (respectivement 28,2 % et 23,0 % chez les mères versus 16,4 % et 10,8 % chez les pères). Le niveau universitaire était de 15,3 % chez les mères versus 47,0 % chez les pères (Tableau I).

**Tableau I:** Characteristics of students interested in specialisation (n = 287) Caractéristiques des étudiants désireux de faire une spécialité (n = 287)

Caractéristiques et facteurs	n (%)
Sexe féminin	123 (42,8)
État civil	
célibataire	276 (96,2)
en couple	11 (3,8)
Activité génératrice de revenu	27 (9,4)
Situation parentale	
2 parents vivants	200 (69,6)
1 parent décédé	80 (27,9)
2 parents décédés	7 (2,5)
Éducation mère	
non scolarisée	81 (28,2)
niveau primaire	66 (23,0)
niveau secondaire	96 (33,4)
niveau universitaire	44 (15,3)
Éducation père	
non scolarisé	47 (16,4)
niveau primaire	31 (10,8)
niveau secondaire	74 (25,8)
niveau universitaire	135 (47,0)
Désir de passer le concours d’internat	218 (75,9)
Milieu d’exercice	231 (80,5)
urbain	
rural	10 (3,5)
à l’étranger	46 (16,0)
Type d’exercice	33 (11,5)
privé	
public	34 (11,8)
mixte	220 (76,7)
Aspiration au travail à temps	
plein	67 (22,3)
partiel	220 (76,7)

**Figure 1:**
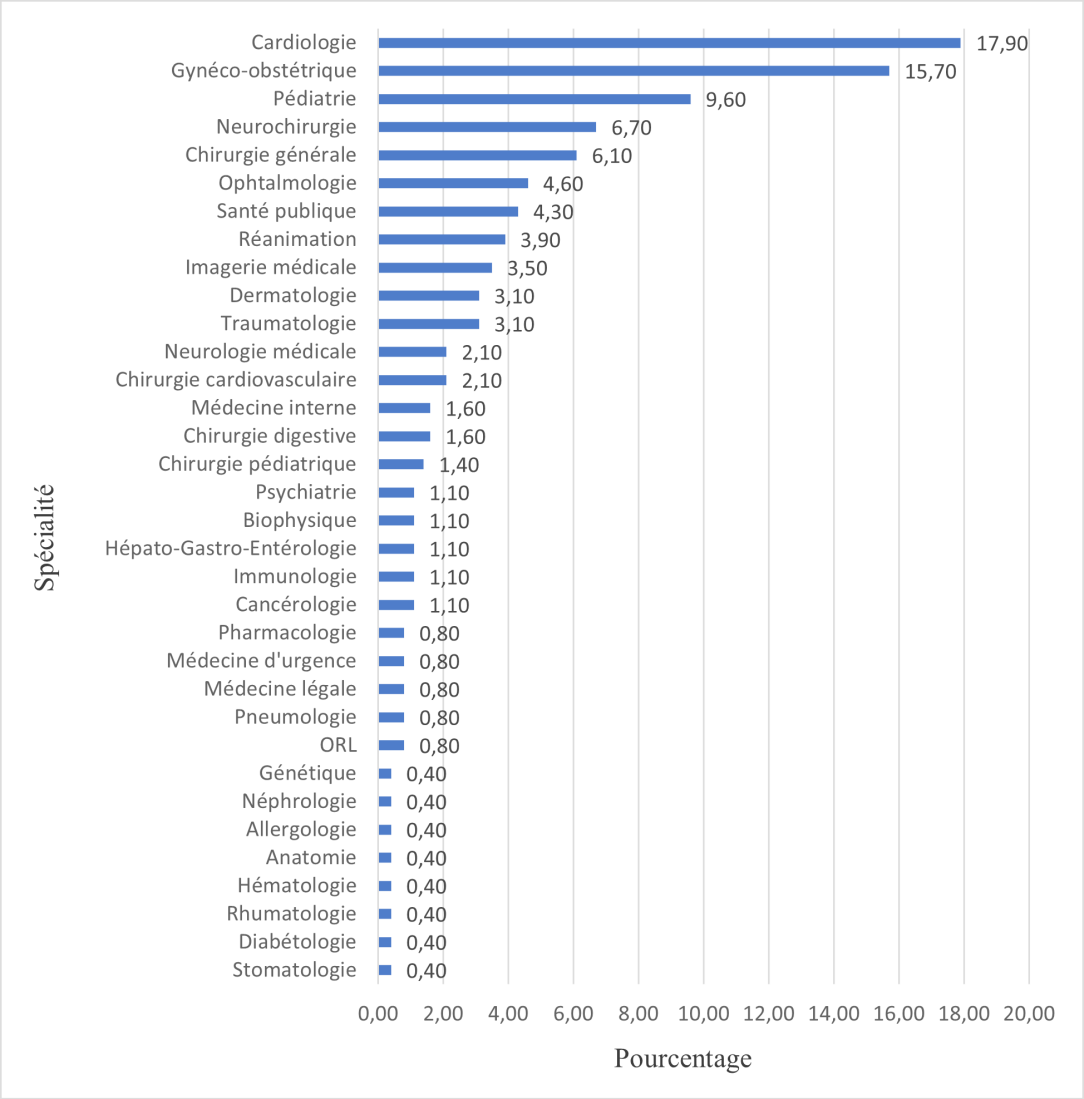
Répartition des choix de spécialité Distribution of speciality choices

Les 3 disciplines de spécialité les plus choisies étaient: la cardiologie (17,9 %), la gynéco-obstétrique (15,7 %) et la pédiatrie (9,6 %) (Fig. 1).

Le désir de passer le concours d’internat était plus fréquent chez les étudiants ayant opté pour une spécialité chirurgicale. Le choix d’une spécialité médicale était plus influencé par le désir de travailler à temps partiel (Tableau II).

**Tableau II:** Analyse comparative des facteurs personnels et sociodémographiques selon le choix de spécialité Comparative analysis of personal and socio-demographic factors according to speciality choice

Variables et modalités (%)	Spécialité médicale (n =166)	Spécialité chirurgicale (n=121)	p
Sexe féminin	40,4	46,3	0,33
Célibataire	95,2	97,5	0,25
Niveau d’étude de la mère			
non scolarisée	26,5	30,5	0,46
primaire	23,5	22,3	
secondaire	36,1	29,7	
universitaire	13,9	17,5	
Niveau d’étude du père			
non scolarisé	16,2	16,5	0,30
primaire	7,8	14,8	
secondaire	27,1	24,0	
universitaire	48,9	44,7	
Désir de passer l’internat	71,1	82,5	0,02
Milieu d’exercice			
urbain	78,9	82,7	0,24
rural	3,5	3,3	
à l’étranger	17,5	14,0	
Activité génératrice de revenus	10,2	7,4	0,27
Type d’exercice			
privé	11,4	11,6	0,26
public	11,4	12,4	
mixte	77,1	76,0	
Aspiration au travail à temps partiel	80,7	71,1	0,04

Les étudiants ayant choisi une spécialité médicale étaient plus guidés par l’engagement social que ceux ayant choisi une spécialité chirurgicale. Par contre, ces derniers étaient plus influencés par le prestige auprès des collègues et les résultats immédiats après intervention (Tableau III).

**Tableau III:** Analyse comparative des facteurs d’influence selon le choix de spécialité Comparative analysis of influencing factors according to speciality choice

		Spécialité Médicale (n =166) Moyenne ± écart-type	Spécialité chirurgicale (n=121) Moyenne ± écart-type	p
Style de vie médicale				
Q1	Horaire de garde acceptable	2,7 ± 1,5	2,6 ± 1,6	0,72
Q2	Heures de pratique acceptables	3,1 ± 1,5	2,9 ± 1,6	0,66
Q3	Choisir ce que je veux faire en médecine	3,9 ± 1,5	4,0 ± 1,4	0,79
Q4	Faire d’autres choses en dehors de la médecine	3,1 ± 1,6	3,1 ± 1,7	0,74
Orientation sociétale				
Q5	Se laisser des ouvertures	3,8 ± 1,4	3,6 ± 1,3	0,17
Q6	Se concentrer sur des patients de la communauté	3,5 ± 2,3	3,8 ± 1,6	0,18
Q7	Relations à long terme avec les patients	2,6 ± 1,6	2,7 ± 1,2	0,66
Q8	Guidé par l’engagement social	3,4 ± 1,6	3,0 ± 1,6	0,04
Q9	Intérêt et pratique de la promotion de la santé	3,7 ± 1,5	3,5 ± 1,4	0,20
Prestige				
Q10	Besoin de pratique lucrative dû aux dettes	1,8 ± 1,4	1,7 ± 1,3	0,45
Q11	Potentiel de revenu élevé indépendamment des dettes	2,6 ± 1,5	2,5 ± 1,6	0,48
Q12	Prestige auprès des collègues	2,5 ± 1,6	2,9 ± 1,6	0,04
Q13	Activité concentrée sur les soins hospitaliers	3,3 ± 1,4	3,4 ± 1,5	0,80
Q14	Résultats immédiats des interventions	3,0 ± 1,5	3,5 ± 1,4	0,01
Structure de soins				
Q15	Activité concentrée sur les soins d’urgence	3,1 ± 1,5	3,3 ± 1,5	0,40
Q16	Grande diversité de problèmes et de patients de tous âges	3,1 ± 1,4	3,0 ± 1,5	0,56
Q17	Moins grande diversité de problèmes et patients d’âge précis	2,4 ± 1,5	2,3 ± 1,4	0,72
Q18	Plus de chance de pratique dans un grand centre urbain	3,4 ± 1,4	3,3 ± 1,5	0,64
Q19	Plus de chance de pratique dans un centre rural	1,9 ± 1,2	2,0 ± 1,4	0,81
Q20	Formation de courte durée	1,8 ± 1,2	1,6 ± 1,2	0,22
Influence de la formation				
Q21	Formation difficile mais stimulante	3,3 ± 1,5	3,6 ± 1,5	0,17
Q22	Identification à un médecin de la même spécialité	3,5 ± 1,5	3,4 ± 1,7	0,83
Q23	Préparation au concours d’internat	2,1 ± 1,4	2,1 ± 1,3	0,81
Q24	Forte influence des stages d’externat	3,5 ± 1,6	3,1 ± 1,7	0,09

Les facteurs Q3 (Choisir ce que je veux faire en médecine) et Q5 (Se laisser des ouvertures) étaient globalement les plus influents (Tableau III), mais ne différaient pas entre les 2 groupes « spécialité médicale » et « spécialité chirurgicale ».

## Discussion

Les facteurs d’influence des choix de carrière ont fait l’objet de plusieurs travaux avec différents types de questionnaires [6, 11, 13]. Celui de Beaulieu se distingue par la validation de sa version française et par sa relative simplicité applicable au système éducatif ivoirien [3].

Le premier constat de cette étude est la forte proportion d’étudiants désireux de faire une spécialité. Sur un effectif de 297 étudiants, seuls 10 n’avaient pas le projet de faire une spécialité après leur thèse. Ce constat peut s’expliquer par la non-reconnaissance de la médecine générale comme spécialité en Côte d’Ivoire.

Si le désir des étudiants de faire une spécialité est accru, la réalité après la soutenance de la thèse d’exercice est toute autre. Le secteur public pour l’exercice de la profession est organisé par le ministère de la fonction publique et le ministère de la santé. Le choix du secteur public expose le médecin à une affectation dans un milieu rural dans lequel il n’existe aucune possibilité de formation. Les centres hospitalo-universitaires, situés dans 2 grandes villes (4 à Abidjan et 1 à Bouaké) concentrent la quasi-totalité des spécialistes chargés de l’enseignement dans le cadre des diplômes d’études spécialisés.

Les étudiants ayant été admis au concours d’internat sont d’emblée affectés dans un centre hospitalo-universitaire et bénéficient donc d’un accès facile à la spécialisation. Ceci explique le fort taux d’étudiants désireux de passer le concours d’internat (75,9 %).

Cette situation n’est évidemment pas comparable au système français pour 2 raisons: le choix de la spécialité est fonction du rang de l’étudiant aux épreuves classantes nationales obligatoires et le nombre de centres hospitaliers universitaires offrant des possibilités de spécialisation est bien plus important en France (32 CHRU) [4].

Les spécialités les plus fréquemment retrouvées étaient la cardiologie (17,9 %), la gynéco-obstétrique (15,7 %) et la pédiatrie (9,6 %). Le choix de la cardiologie comme première spécialité dans notre contexte n’est pas surprenant. L’Institut de Cardiologie d’Abidjan est une structure de référence en Côte d’Ivoire et dans la sous-région ouest-africaine par la qualité de ses prestations avec un équipement de pointe et par son engagement dans une démarche qualité [12]. Il constitue ainsi un cadre propice à la formation des étudiants en pré-doctoral mais aussi en spécialisation. L’étude marocaine de Matrane et al montre des résultats similaires avec ces 3 spécialités classées parmi les 5 premières disciplines choisies par les étudiants [11]. En France, la médecine générale (16,85 %), la pédiatrie (8,14 %), l’anesthésie-réanimation (6,25 %) et la gynécologie-obstétrique (5,52 %) étaient les spécialités les plus prisées en 2019 [16].

En revanche, l’absence de choix de certaines spécialités comme la parasitologie, la bactériologie, la virologie et la biochimie médicale découle des retards d’équipement des laboratoires de la faculté de médecine d’Abidjan après la crise post-électorale de 2011. L’irrégularité des travaux pratiques en laboratoire peut donc expliquer le manque d’engouement pour les spécialités biocliniques au profit des spécialités cliniques.

Le désir de passer l’internat était plus fréquent chez les étudiants ayant choisi une spécialité chirurgicale. Ce constat s’explique par la répartition quasi-exclusive des chirurgiens enseignants dans les centres hospitalo-universitaires. Ces derniers offrent également des possibilités de formations chirurgicales diverses liées à un équipement technique plus performant. Le concours d’internat en Côte d’Ivoire favorise donc l’accessibilité aux spécialités chirurgicales.

Le travail à temps partiel pour le personnel soignant n’est autorisé en Côte d’Ivoire que pour les praticiens hospitaliers enseignants. Il s’agit en fait d’un temps de travail partiel au plan sanitaire car ceux-ci doivent effectuer 20 à 30 heures d’activités de soins par semaine [1], le reste du temps étant dédié aux charges pédagogiques. Le choix d’une spécialité médicale des étudiants était plus influencé par le désir d’un travail à temps partiel. Nos résultats ont globalement montré une forte proportion d’étudiants aspirant à un travail à temps partiel et on notait une forte moyenne du facteur Q5 (Se laisser des ouvertures) (Tableau III). Certaines études l’ont expliqué par la fuite des contraintes professionnelles et le besoin de pouvoir concilier vie professionnelle et vie de famille [6, 11]. À ces deux arguments s’ajoutent les revendications salariales des médecins ivoiriens qui, faisant l’objet de grèves incessantes [5], peuvent également expliquer le désir d’un travail à temps partiel. En effet, les médecins ivoiriens développent de plus en plus d’activités génératrices de revenus (commerce, entreprenariat, etc…) pour compenser leur faible rémunération, mais il existe très peu de données sur ce sujet. Une étude africaine réalisée sur l’ensemble du personnel soignant a montré que 8,46 % des enquêtés avaient une activité génératrice de revenus et que celle-ci était majoritairement représentée par le commerce. La moitié des agents de santé qui ont indiqué exercer une activité rémunératrice en dehors de leur fonction principale y consacraient au minimum 5 heures par semaine [9]. Dans notre étude, environ 1 étudiant sur 10 (9,4 %) avait une activité extra-médicale génératrice de revenus. La forte aspiration au travail à temps partiel chez les étudiants ayant opté pour le choix d’une spécialité médicale peut se justifier par l’inégalité des revenus au profit des spécialités chirurgicales, surtout lorsque l’activité professionnelle est mixte (privée et publique).

Les étudiants ayant choisi une spécialité médicale étaient plus guidés par l’engagement social que ceux ayant choisi une spécialité chirurgicale. Choucair et al avaient déjà montré que les étudiants qui avaient opté pour la valeur « orientation biosociale » du questionnaire de Murdoch choisissaient préférentiellement les carrières favorisant l’interaction médecin-malade, comme les spécialités médicales et les soins primaires. En France, une analyse multivariée a mis en évidence une forte corrélation de l’engagement social avec le choix de la médecine générale [6].

Les choix de spécialités chirurgicales étaient plus influencés par le prestige auprès des collègues et les résultats immédiats après intervention, comme dans la plupart des études [2, 11].

Cette étude présente des limites. Certains facteurs d’influence, et en particulier certains freins au choix d’une spécialité, ont pu ne pas être abordés, d’autant plus que le questionnaire utilisé a été élaboré et validé en Amérique du Nord. Aucune évaluation du niveau de renseignement des étudiants sur les différentes spécialités n’avait été réalisée avant le remplissage du questionnaire. Enfin, un biais de déclaration pouvait persister malgré l’anonymisation du questionnaire.

## Conclusion

En Côte d’Ivoire, le concours d’internat et la spécialisation sont fortement prisés par les étudiants en fin de cursus. La cardiologie, la gynéco-obstétrique et la pédiatrie sont les spécialités les plus choisies. Les facteurs influençant le choix de spécialité sont: le désir de passer le concours d’internat, l’aspiration au travail à temps partiel, l’engagement social, le prestige et les résultats immédiats après intervention.

Les enjeux en termes de démographie médicale en Côte d’Ivoire sont actuellement axés sur une augmentation des professionnels de santé, surtout des médecins, ainsi que sur une meilleure répartition des spécialistes sur le territoire. L’équipement efficient des structures sanitaires pourrait contribuer à une mise en valeur des autres spécialités moins choisies en les rendant plus attractives. Une réorganisation du système avec affectation d’enseignants dans les hôpitaux régionaux avec un minimum d’équipement est indispensable afin de permettre une « décentralisation » du cursus de spécialisation, surtout pour les spécialités chirurgicales.

Une reconnaissance de la médecine générale comme spécialité pourrait augmenter son prestige et sa valorisation. Quant à l’aspiration au travail à temps partiel, elle peut s’expliquer par le besoin de concilier vie familiale et vie professionnelle, mais aussi par un projet parfois inavoué de développer une activité extra-médicale lucrative en vue de compenser une faible rémunération.

## Liens d’intérêts

Les auteurs ne déclarent aucun lien d’intérêt.

## Contribution des auteurs

Marie-Paule Bernadette N’CHO-MOTTOH: conception du protocole, recueil des données, rédaction du manuscrit

Iklo COULIBALY: relecture du manuscrit

Bénédicte BOKA: recueil des données Djenamba BAMBA-KAMAGATE: interprétation des résultats

Arnaud EKOU: analyse statistique

Alain AUBREGE: relecture du manuscrit
